# Assessment of corrosion protection of Ti_3_C_2_T_*x*_@CoAl-LDH composite coatings formed on steel sheets

**DOI:** 10.1039/d5ra09120a

**Published:** 2026-01-28

**Authors:** Lina Huang, Jun Huang, Xiaoguang Liang, Yongsheng Cao

**Affiliations:** a College of Architecture and Civil Engineering, Nanning University Nanning 530200 Guangxi PR China

## Abstract

Multiple kinds of layered materials have acquired much attention in the field of corrosion inhibition due to their distinctive two-dimensional (2D) structure. However, the inherent disadvantages of a single 2D material remarkably limit its performance. In this work, the layered Ti_3_C_2_T_*x*_@cobalt-aluminum layered double hydroxide (Ti_3_C_2_T_*x*_@CoAl-LDH) heterostructures have been synthesized successfully, and the obtained Ti_3_C_2_T_*x*_@CoAl-LDH composite integrates the advantages of the high electron conductivity of Ti_3_C_2_T_*x*_ and the high electro-chemical activities of CoAl-LDH, thus effectively enhancing the electrochemical reactivity of electrode materials and accelerating the kinetics of the Faraday reaction. Additionally, the functional Ti_3_C_2_T_*x*_ with different functional groups enables CoAl-LDH to exhibit improved corrosion resistance performance. The corrosion resistance performance is appraised by the Tafel curve and electrochemical impedance spectroscopy. The results of the electrochemical tests demonstrate that the Ti_3_C_2_T_*x*_@CoAl-LDH composite delivers excellent corrosion resistance performance with the lowest corrosion current density (2.8338 × 10^−9^ A cm^−2^) and the largest corrosion resistance (1.656 × 10^5^ Ω). This work paves the way for the potential application of Ti_3_C_2_T_*x*_-based materials in the field of corrosion protection.

## Abstract 2

Multiple kinds of layered materials have acquired much attention in the field of corrosion inhibition due to their distinctive two-dimensional (2D) structure.

## Graphical abstract

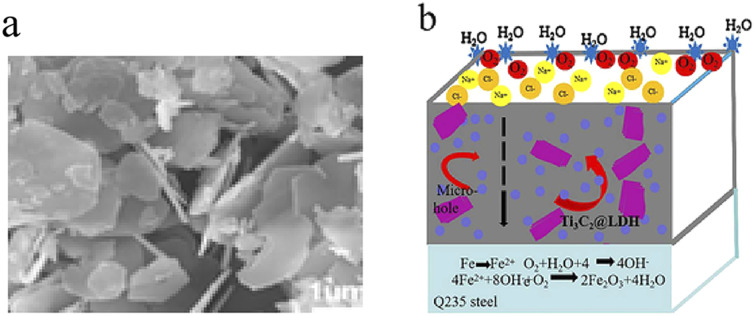

## 1. Introduction

Because of its excellent mechanical properties, steel is an indispensable metal alloy in industrial construction. However, its poor corrosion resistance significantly shortens its service life in applications such as reinforced concrete infrastructure, especially in coastal environments. As chloride ions penetrate the structure, the steel reinforcement begins to corrode, which can easily lead to the failure of reinforced concrete and may even cause collapse before reaching its expected service life. These issues raise serious concerns regarding economic losses, disruption of social functions, and the wastage of energy and resources. Therefore, there is an urgent need to develop methods to enhance the corrosion resistance of steel substrates.^1–3^

Coatings or protective films represent an effective strategy. Due to their straightforward preparation, effective physical barrier properties, and even certain self-healing capabilities,^4^ they are commonly used to protect metals from chloride-induced corrosion. Examples include the use of epoxy resins, hybrid inhibitors with rust suppressants,^5,6^ microencapsulation,^7–10^ and other techniques to delay the penetration of corrosive media.^11,12^ Although these approaches demonstrate good anti-corrosion performance, they often introduce a weak bonding interface between the coating and the cementitious matrix—a critical concern since steel reinforcement is embedded within concrete structures. Consequently, growing research attention is being directed toward inorganic films on metal substrates for corrosion protection owing to their environmentally friendly characteristics.^13–15^

Layered double hydroxide (LDH) possesses a unique layered structure similar to that of brucite (Mg(OH)_2_). Its general chemical formula is [M^2+^_1−*x*_M^3+^(OH)_2_]^*x*+^(A^*n*−^)_*x*/*n*_·*m*H_2_O,where M^2+^ and M^3+^ represent divalent and trivalent cations, respectively, A^*n*−^ is an interlayer anion, and x typically ranges from 0.20 to 0.33.^16–20^ The interlayer anions in LDH are weakly bonded to the brucite-like layers *via* hydrogen bonds and can be readily exchanged with other anions that interact electrostatically with the main layers, giving LDH good ion-exchange capacity and structural tunability.^21–24^ Moreover, its layered structure with low surface energy and inherent surface roughness contributes to hydrophobicity and water resistance.

MXene(Ti_3_C_2_T_*x*_), a transition metal carbide nanomaterial, is a type of two-dimensional inorganic compound with a graphene-like structure.^25–27^ It typically exhibits an accordion-like morphology, and its surface can be functionalized with various groups, endowing it with high thermal conductivity, excellent electrical conductivity, and favorable mechanical properties. Furthermore, its large specific surface area and distinctive layered structure make it highly promising for corrosion protection applications.^28,29^ Recently, a thin dodecyltrimethoxysilane/Ti_3_C_2_T_*x*_ coating was fabricated on an aluminium alloy *via* a green electrodeposition method. By combining dodecyltrimethoxysilane with Ti_3_C_2_T_*x*_, the coating demonstrated a low corrosion current density (9.284 × 10^−7^ A cm^−2^) in a 0.5 M H_2_SO_4_ + 2 ppm HF solution and a low wear rate (3.82 × 10^−3^ mm^3^ N^−1^ m^−1^). Such thin MXene coatings, which can be applied at a high rate, show potential for use in proton exchange membrane fuel cells (PEMFCs).^30^ Additionally, the metallic conductivity, excellent barrier properties, and structural versatility with rich surface terminations allow MXene nanosheets to be applied for surface protection of metallic bipolar plates in PEMFC environments, while also reducing interfacial contact resistance and enhancing coating-substrate adhesion.^30^ Thus, MXenes are promising candidates for improving adhesion in corrosion protection applications.

In light of the above research progress, this work explores a feasible route to enhance corrosion protection by combining the intrinsic properties of Ti_3_C_2_T_*x*_ with the excellent waterproof functionality of CoAl-LDH. Moreover, since both Ti_3_C_2_T_*x*_ and LDH possess two-dimensional layered structures, their integration is expected to result in a denser layered architecture, with structural modifications likely leading to performance improvements.^31^ The anti-corrosion performance of the composite coating was evaluated through electrochemical tests, which reveal outstanding corrosion protection capability of the Ti_3_C_2_T_*x*_@CoAl-LDH composite. Finally, the corrosion protection mechanism of the composite coating was analyzed. Electrochemical results demonstrate that the Ti_3_C_2_T_*x*_@CoAl-LDH composite exhibits exceptional corrosion resistance, with a corrosion current density as low as 2.8338 × 10^−9^ A cm^−2^ and a charge transfer resistance of 1.656 × 10^5^ Ω cm^2^. This work paves the way for the potential application of Ti_3_C_2_T_*x*_-based materials in the field of corrosion protection and offers a new strategy for achieving high-performance anti-corrosion coatings based on Ti_3_C_2_T_*x*_ nanocomposites.

## 2. Experiments

### 2.1. Materials

Urea (H_2_NCONH_2_, purity 99%), cobalt nitrate hexahydrate (Co(NO_3_)_2_·6H_2_O, purity 99%), aluminium nitrate hexahydrate (Al(NO_3_)_3_·9H_2_O, purity 99%) and sodium chloride (NaCl) were obtained from Guangdong Guanghua Sci-Tech Co., Ltd, China. Sodium tungstate (Na_2_WO_4_·2H_2_O), waterborne resin epoxy (H228A—90% Bisphenol A Epoxy Resin + 10% AGE; the dielectric constant range is usually between 2.5 and 3.5; the main functional groups include alcohol groups (–OH), ether groups (–O–), and ester groups (–COO–)), and hardener (H228B; with a dielectric constant between 2 and 3; with the main functional groups of alcohol groups (–OH), ether groups (–O–), and ester groups (–COO–); with the ratio of the resin epoxy to the hardener is 1 : 2) were purchased from Guangdong Shantou Xilong Sci-Tech Co., Ltd, China. Of which, sodium tungstate was introduced as a corrosion-inhibiting agent. Its anion is intended to be intercalated into the LDH structure, providing active corrosion protection through an inhibitory release mechanism.

Ti_3_C_2_T_*x*_ material was obtained from Jiangsu Xianfeng Sci-Tech Co., Ltd, in China. Deionized water was used in aqueous solutions and filtration. All the chemical reagents applicable to this experiment are of analytical grade (AR) without further purification.

### 2.2. Pretreatment of Ti_3_C_2_T_*x*_ powder

0.5 g of Ti_3_C_2_T_*x*_ powder was added to 100 mL of DI water, and the mixture was exfoliated *via* probe sonication using a “Qsonica Q700” ultrasonicator (500 W, 20 kHz) for 1 hour while cooling in an ice-water bath. The solution was centrifuged at 3500 rpm, and the well-dispersed Ti_3_C_2_T_*x*_ solution was obtained.

### 2.3. Preparation of Ti_3_C_2_T_*x*_@CoAl-LDH composite films

The raw CoAl–CO_3_^2−^-LDH film was prepared by the urea hydrolysis method. First, 2.62 g of Co(NO_3_)_2_·6H_2_O and 1.69 g of Al(NO_3_)_3_·9H_2_O were dissolved in 100 mL of deionized water in a three-necked flask, and the above Ti_3_C_2_T_*x*_ solution was added into the mixed solution and stirred magnetically until a uniform mixture was obtained. Then, 50 mL of Na_2_WO_4_·2H_2_O solution was added drop by drop, and the pH was adjusted to 10 with the addition of NaOH solution and maintained for 12 h at 80 ^°^C under N_2_ gas atmosphere. Finally, the obtained solution was centrifuged and cleaned with DI water and dried using a freeze-drying method.

### 2.4. Pretreatment of steel substrate

The base material used in this article was a Q235 steel substrate. The size of the Q235 steel substrate was 1 mm × 1 mm. First, the steel substrate was ultrasonically cleaned in absolute ethyl alcohol for 5 min to remove the grease on the surface and filtered with deionized water repeatedly; then, the sample was polished with SiC sandpaper in the same direction to remove the defects (such as scratches, dents). Then, acetone and deionized water were used for ultrasonic cleaning for 5 minutes to further clean the surface of the sample. 0.15 g of the Ti_3_C_2_T_*x*_@CoAl-LDH composite obtained in the step mentioned in Section 2.3 was added to 24 g of DI water and sonicated for 1 h under a N_2_ gas atmosphere; subsequently, 10 g of epoxy resin and 20 g of hardener were mixed in the above dispersed solution. Last, the absorber slurry was uniformly deposited *via* spin-coating at 1500 rpm for 60 s using a WS-650MZ-23NPP spin coater to control the initial wet film thickness. Then, the coated samples were dried at 60 °C for 12 hours in a vacuum oven to remove solvents and form a stable film. Then, the thickness of the coating is approximately 3–3.5 µm, according to SEM images. Additionally, adhesion was evaluated *via* cross-cut tape tests (ASTM D3359), and the coating achieved a Class 4B rating (excellent adhesion, <5% removal).

For convenience, it is denoted as Ti_3_C_2_T_*x*_@CoAl-LDH, and the raw steel sheet is denoted as the blank sample, and CoAl-LDH is denoted as the raw CoAl-LDH sample.

### 2.5. Characterizations

X-ray diffraction pattern and Fourier transform infrared spectrophotometer (FT-IR spectra, Nicolet iS5, Thermo, USA) were used to analyze the composition and chemical structures of Ti_3_C_2_T_*x*,_ CoAl-LDH and Ti_3_C_2_T_*x*_@CoAl-LDH composite. The microstructure and morphology of Ti_3_C_2_T_*x*,_ CoAl-LDH and Ti_3_C_2_T_*x*_@CoAl-LDH composite were evaluated by field-emission scanning electron microscopy (FE-SEM) and high-resolution transmission electron microscopy (HRTEM).

Electrochemical impedance spectroscopy (EIS) was employed to measure the corrosion behavior of the steel substrate and its corrosion resistance. EIS was carried out in a three-electrode cell with a mercury oxide electrode (MOE), a platinum plate counter electrode and the sample as the working electrode. Before starting the test, each sample was soaked in the electrolyte solution for 30 minutes to stabilize the system. In the frequency range of 10 KHz–10 MHz, the perturbation amplitude was 5 mv. First, the sample was soaked in a saturated Ca(OH)_2_ solution for 10 days to simulate the passivation conditions of the steel substrate in concrete to obtain the EIS spectra. Then, the simulated pore solution containing 3.5 wt% NaCl solution was regarded as the electrolyte for electrochemical and immersion experiments. For the potentiodynamic polarization test, the potential scan range was ±500 mV with respect to OCP (open circuit potential) and the potential scan rate was 5 mV s^−1^. Corrosion potential (*E*_corr_) and corrosion current density (*I*_corr_) were obtained by Tafel extrapolation technique. All electrochemical tests were performed on three parallel samples to ensure the reproducibility of the results.

## 3. Results and discussions

### 3.1. Characterizations of Ti_3_C_2_T_*x*_@CoAl-LDH composite

**Fig. 1:**
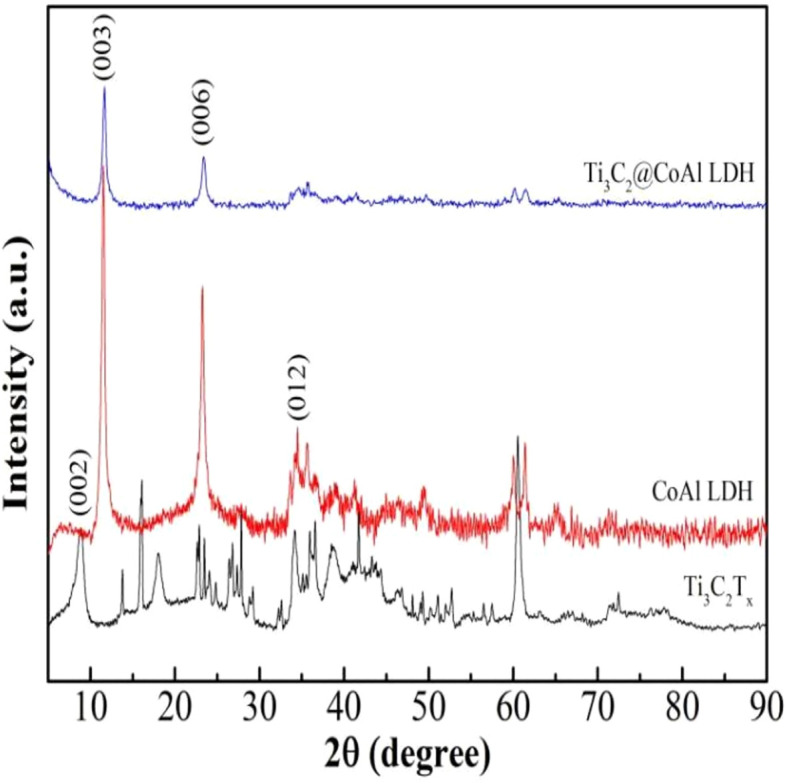
XRD patterns of raw Ti_3_C_2_T_*x*_, CoAl-LDH and the Ti_3_C_2_T_*x*_@CoAl-LDH composite.

Fig. 1 shows the X-ray diffraction patterns. The main characteristic diffraction peaks of the XRD pattern indicate that the phase is CoAl-LDH, which exhibits identical peaks corresponding to the (003)/(006) reflections at 2*θ* = 11.46°/22.80° of the layered CoAl-LDH, denoting that the nano-crystallized LDH was smoothly obtained using the urea hydrolysis method. Among these, the typical (002) peak of Ti_3_C_2_T_*x*_ cannot be detected by the XRD technique due to its small amount which cannot be detected.

**Fig. 2:**
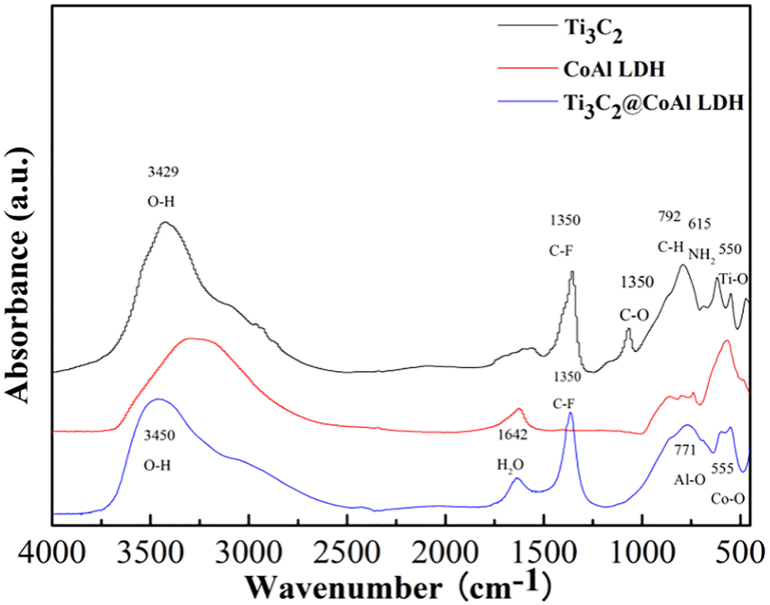
FT-IR spectra of raw Ti_3_C_2_T_*x*_, CoAl-LDH and the Ti_3_C_2_T_*x*_@CoAl-LDH film.

In addition, the Fourier-transform infrared (FT-IR) spectra of pristine Ti_3_C_2_T_*x*_, pristine CoAl-LDH, and the Ti_3_C_2_T_*x*_@CoAl-LDH composite are illustrated in Fig. 2. The spectrum of the composite integrates the characteristic bands of both precursors, while exhibiting new vibrational modes at specific wavenumbers, confirming the successful hybridization of Ti_3_C_2_T_*x*_ with CoAl-LDH and the possible surface functionalization. A detailed analysis is as follows: broad absorption peaks appear at 3450 cm^−1^ and 1642 cm^−1^, which are due to the stretching vibration of the hydrogen-bonded hydroxyl groups (–OH) and the deformation vibration of water molecules, respectively. The low wavenumber bands at 555 cm^−1^ and 700 cm^−1^ are caused by the vibration of Co–O and Al–O bonds, respectively. Compared with raw CoAl-LDH and raw Ti_3_C_2_T_*x*_ powder, Ti_3_C_2_T_*x*_@CoAl-LDH composite has remained 1350 cm^−1^ which is due to the stretching vibration of C–F and 792 cm^−1^ which is due to the stretching vibration of C–H and 615 cm^−1^ which could be attributed to –NH_2_ stretching vibration derived from the addition of Ti_3_C_2_T_*x*._ The appearance of the amino groups means that the Ti_3_C_2_T_*x*_@CoAl-LDH composite has been functionalized by the introduction of Ti_3_C_2_T_*x*_ powder.^32^ The FT-IR spectra of raw Ti_3_C_2_T_*x*_, CoAl-LDH and the Ti_3_C_2_T_*x*_@CoAl-LDH composites are illustrated in Fig. 2.

**Fig. 3:**
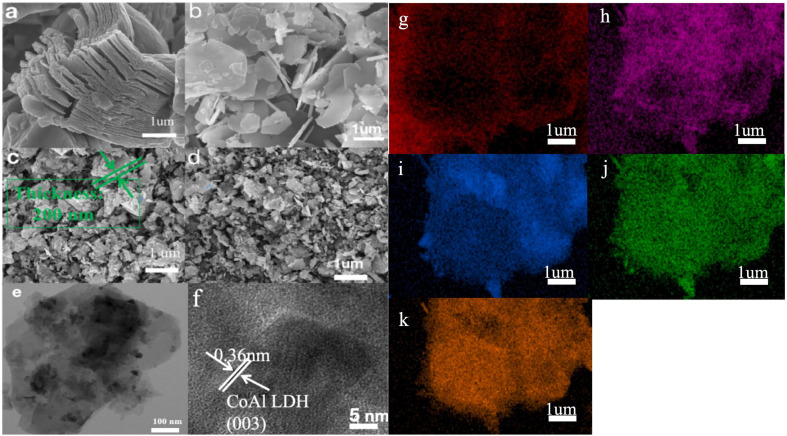
(a) SEM images of Ti_3_C_2_T_*x*_, (b) CoAl-LDH, (c and d) and the Ti_3_C_2_T_*x*_@CoAl-LDH composite; (e and f) TEM and HRTEM images of the Ti_3_C_2_T_*x*_@CoAl-LDH composite; and (g–k) the elemental mapping of C, O, Ti, Al, and Co elements, respectively.

The morphology and microstructure of raw Ti_3_C_2_T_*x*_ powder, raw CoAl-LDH and Ti_3_C_2_T_*x*_@CoAl-LDH composite were characterized by SEM and TEM. The SEM observations of pristine Ti_3_C_2_T_*x*_, CoAl-LDH, and Ti_3_C_2_T_*x*_@CoAl-LDH composite were displayed in Fig. 3(a–d). Fig. 3(a) demonstrated an accordion-shaped layer nanostructure, which is of raw Ti_3_C_2_T_*x*_ material, and Fig. 3(b) showed the structure of raw CoAl-LDH without modification by Ti_3_C_2_T_*x*_. These two kinds of 2D materials typically appear layered, with a certain spacing and connectivity between layers. Additionally, due to the topography and structural characteristics of the material surface, protrusions, depressions, or textures may be observed on the layer surface. These shapes and structural features can be used to characterize and analyze the material, thereby understanding its microstructure and composition.

After the formation of Ti_3_C_2_T_*x*_@CoAl-LDH nanohybrids, Fig. 3(c and d) showed the SEM images of the Ti_3_C_2_T_*x*_@CoAl-LDH sample on the surface of the steel substrate. It can be clearly seen that the sample exhibits an aggregated sheet-like structure which did not keep the original crystal structure of raw CoAl-LDH with an evident 2D sliced structure and Ti_3_C_2_T_*x*_ with an accordion-shaped structure, and the typical LDH crystal indicates the successful synthesis of LDH in the steel substrate. According to (Fig. 3(c)), we have measured the thickness of the composite Ti_3_C_2_T_*x*_@CoAl-LDH, and the thickness of the coating is between 100 nm and 300 nm.

For further analysis of the structure and phase, TEM and HRTEM images are shown in Fig. 3(e and f). The good crystallinity characteristics of CoAl-LDH were confirmed by the measured interplanar distance of 0.36 nm, which is well matched with the (003) crystallographic plane of CoAl-LDH (Fig. 3(f)). Simultaneously, the raw accordion shape of Ti_3_C_2_T_*x*_ powder could not be examined from the HRTEM images. Meanwhile, Fig. 3(g and h) demonstrate that the elements of Ti, C, O, Co and Al were fairly uniformly dispersed across the SEM image, which indicates that Ti_3_C_2_T_*x*_ and CoAl-LDH were successfully coupled, although the element Ti exhibited a very small amount, and the elements Co and Al were unevenly dispersed, which was verified by elemental mapping as shown in Fig. 3(e–g). The inhomogeneous spatial distribution of Ti, C, O, Co, and Al elements, as revealed in Fig. 3(g and h), suggests a complex microstructure within the coating. This heterogeneity is not merely morphological but may have significant electrochemical implications. Specifically, local compositional variations can create micro-galvanic cells, where regions with nobler elements (*e.g.*, Co- or Ti-rich zones) act as cathodes, and adjacent areas with less noble or more active elements (*e.g.*, Al-rich or porous carbon-rich zones) act as anodes. In the presence of an electrolyte, these localized corrosion microcells could preferentially drive the dissolution of the anodic regions, potentially initiating pitting or accelerating localized degradation. Therefore, achieving a more homogeneous elemental distribution should be a target for further optimization of the coating's long-term corrosion protection.

### 3.2. Corrosion resistance of the Ti_3_C_2_T_*x*_@CoAl-LDH nanocomposite

**Fig. 4:**
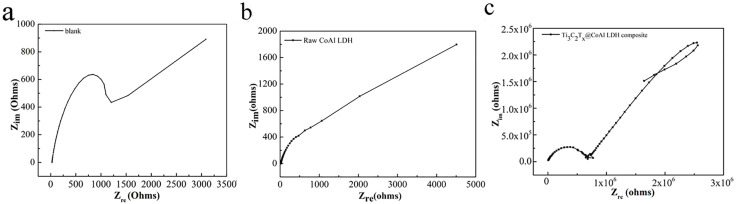
Nyquist results of the (a) blank sample; (b) raw CoAl-LDH sample; and (c) Ti_3_C_2_T_*x*_@CoAl-LDH composite.

**Fig. 5:**
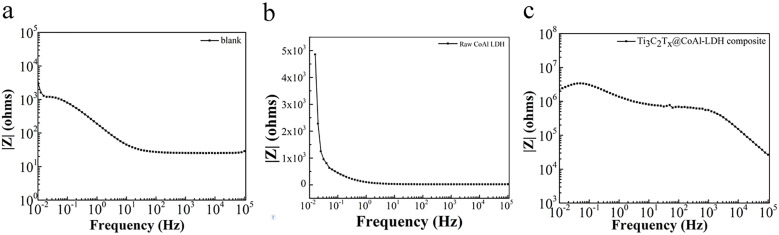
Bode plots of the (a) blank sample; (b) raw CoAl–LDH sample; and (c) Ti_3_C_2_T_*x*_@CoAl-LDH composite.

**Fig. 6:**
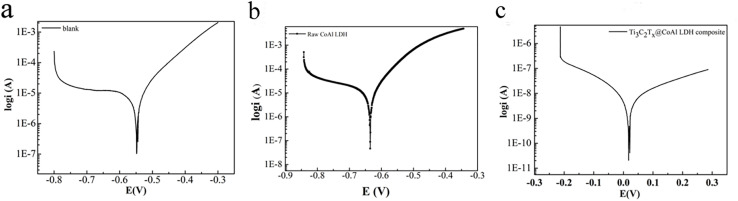
Tafel curve of the (a) blank sample. The curve shown is a representative plot. The average *E*_corr_ from all replicates is −0.55 V *vs.* SCE (see Table 1). Tafel curves of the (b) raw CoAl-LDH sample and (c) Ti_3_C_2_T_*x*_@CoAl-LDH composite.

In order to evaluate the corrosion resistance performance of the composite under three fabrication conditions, the EIS measurements and Tafel curves were performed. Nyquist, Bode and Tafel curves related to different samples immersed in saline solutions are illustrated in in Fig. 4–6, respectively. The protective performance of the coatings can be preliminarily characterized by the impedance arc semicircle diameter in the Nyquist plot. The Ti_3_C_2_T_*x*_@CoAl-LDH coating exhibited a higher |*Z*| value than the raw CoAl-LDH sample, and this indicates higher inhibition of charge transfer between the steel sheet and the prepared film, mainly due to the presence of a dense film that separates the vulnerable metal substrate from corrosive species to a large extent. It is usually identified that in the Bode curves of impedance modulus *vs. f*, the low-frequency impedance value at 0.01 Hz is widely used to appraise the overall anti-corrosion performance of the coatings, and the high-frequency domain impedance modulus is related to the properties of the measured film.^33^

The coating of Ti_3_C_2_T_*x*_@CoAl-LDH composite exhibited a higher |Z| value than the raw CoAl-LDH composite (blank sample), and it indicates higher inhibition of charge transfer between the steel substrate and the prepared film, mainly due to the presence of a dense film that separates the vulnerable metal substrate from corrosive species to a large extent, which can be contributed to the emergence of a steady passive coating.^33^ The maximum |*Z*|_0.01Hz_ value for the Ti_3_C_2_T_*x*_@CoAl-LDH sample indicated that it has the most favorable corrosion protection among the acquired films.^34–36^ The curves were drawn to derive the parameters of corrosion such as corrosion current density (*I*_corr_), potential (*E*_corr_), polarization resistance (*R*_p_), anodic slope (*β*_a_), cathodic slope (*β*_c_) and corrosion rate (CR), which were obtained by fitting the Tafel plots and are listed in Table 1. The corrosion rate is calculated based on the following equation:

1 CR = *M* × *I*_corr_/*nFρ* × 87 600

wherein *M* denotes the molecular weight of Fe (56), *n* is the Fe chemical valence, *ρ* is the density of the Q235 steel electrode (7.85 g cm^−3^), and F is the Faraday constant (26.8 A h mol^−1^). Additionally, the “Efficiency” values in Table 1 represent the corrosion inhibition efficiency (IE, %). They were calculated using the corrosion rate (CR) data obtained from Tafel extrapolation, according to the following standard formula:

2 IE (%) = [(CR_0_ − CR_1_)/CR_0_] × 100.

**Table 1:** Calculation parameters obtained from fitting the Tafel curves

Samples	*E*_corr_ (V)	*I*_corr_ (A cm^−2^)	*R*_p_ (Ω cm^2^)	*β*_a_	*β*_c_	Efficiency (%)
Q235 steel	−0.55	3.23 × 10^−4^	11 × 10^0^	2.26	1.58	—
CoAl-LDH	−0.63518	1.6624 × 10^−5^	3.051 × 10^3^	83.282	461.69	94.85
Ti_3_C_2_T_*x*_@CoAl-LDH	0.017806	2.8338 × 10^−9^	1.656 × 10^5^	121.35	90.1	99.99

The raw CoAl-LDH and Ti_3_C_2_T_*x*_@CoAl-LDH composite presented an *I*_corr_ of 1.6624 × 10^−5^ and 2.8338 × 10^−9^, and the Ti_3_C_2_T_*x*_@CoAl-LDH composite was nearly five orders of magnitude lower than that of the blank sample. Also, the *E*_corr_ exhibited a positive shift for the Ti_3_C_2_T_*x*_@CoAl-LDH composite in comparison with the raw CoAl-LDH sample. Furthermore, the values of *R*_p_ displayed the same trend, which verifies that the Ti_3_C_2_T_*x*_@CoAl-LDH composite significantly enhanced the corrosion resistance protection. The excellent corrosion resistance function was attributed to the enhanced compactness and barrier properties resulting from the addition of Ti_3_C_2_T_*x*_, and thus decreasing the exposed surface area which can be attacked by the corrosive media. Therefore, the Ti_3_C_2_T_*x*_@CoAl-LDH composite not only decelerates the permeation of corrosive medium but also hinders the diffusion of corrosive products.

Above all, the corrosion parameters, including corrosion current density (*I*_corr_), potential (*E*_corr_), polarization resistance (*R*_p_), anodic slope (*β*_a_), cathodic slope (*β*_c_), and corrosion rate (CR), were obtained by fitting the Tafel plots. These parameters are summarized in Table 1. The corrosion rate was calculated using the following equation:CR = M × *I*_corr_/*nFρ* × 87 600where *M* is the molecular weight of Fe (56), *n* is the Fe chemical valence, *ρ* is the density of the Q235 steel electrode (7.85 g cm^−3^), and F is the Faraday constant (26.8 A h mol^−1^). The raw CoAl-LDH and Ti_3_C_2_T_*x*_@CoAl-LDH composite exhibited corrosion current densities of 1.6624 × 10^−5^ and 2.8338 × 10^−9^ A cm^−2^, respectively. The Ti_3_C_2_T_*x*_@CoAl-LDH composite demonstrated a nearly five-order of magnitude reduction in corrosion current density compared to the blank sample. Additionally, the *E*_corr_ potential of the Ti_3_C_2_T_*x*_@CoAl-LDH composite shifted positively compared to the raw CoAl-LDH sample. The *R*_p_ values showed the same trend, indicating that Ti_3_C_2_T_*x*_@CoAl-LDH significantly improved the corrosion resistance. This excellent corrosion resistance function was attributed to the enhanced compactness and barrier properties provided by Ti_3_C_2_T_*x*_, which decreased the exposed surface area that could be attacked by corrosive media. Therefore, Ti_3_C_2_T_*x*_@CoAl-LDH not only slows the permeation of corrosive media but also impedes the diffusion of corrosive products.

Above all, a larger diameter of the capacitive arc corresponds to a higher charge transfer resistance (*R*_ct_), indicating a more effective barrier against corrosive species and slower corrosion kinetics. For instance, the Ti_3_C_2_T_*x*_@CoAl-LDH composite exhibits the largest arc diameter, which both visually and quantitatively signifies its superior interfacial resistance. Complementarily, the Bode plots offer insights into the coating's capacitive and barrier properties. The high impedance modulus at low frequency (|*Z*|_0.01_) confirms excellent barrier performance, while the broad, high phase angle plateau at intermediate frequencies is characteristic of a dense, capacitor-like protective layer. A shift of this plateau toward lower frequencies, as observed for the Ti_3_C_2_T_*x*_@CoAl-LDH composite, indicates a more stable and less defective interface, thereby retarding electrolyte penetration. The large capacitive arc in Nyquist plots, the high |*Z*| and broad phase angle plateau in Bode plots, and the noblest corrosion potential (*E*_corr_) combined with the lowest corrosion current density (*I*_corr_) all denote that the Ti_3_C_2_T_*x*_@CoAl-LDH composite provides the most effective and durable corrosion protection among all samples tested. This multi-faceted and consistent evidence strongly reinforces the reliability of our performance assessment.

The excellent corrosion resistance of the Ti_3_C_2_T_*x*_@CoAl-LDH composite can be attributed to the enhanced compactness and barrier properties provided by Ti_3_C_2_T_*x*_, which reduce the exposed surface area that can be attacked by corrosive media. In addition, the shift of the corrosion potential from the Tafel plot indicates that the Ti_3_C_2_T_*x*_@CoAl-LDH composite has better corrosion resistance, which may be attributed to the improved polarization performance of the electrode caused by the addition of Ti_3_C_2_T_*x*_, leading to greater stability in corrosive environments. The change in the polarization resistance (*R*_p_) value further confirms this trend, indicating that Ti_3_C_2_T_*x*_@CoAl-LDH composite significantly enhances corrosion resistance. Therefore, Ti_3_C_2_T_*x*_@CoAl-LDH not only slows the permeation of corrosive media but also impedes the diffusion of corrosive products, making it a promising material for practical applications with excellent anti-corrosion performance.

**Fig. 7:**
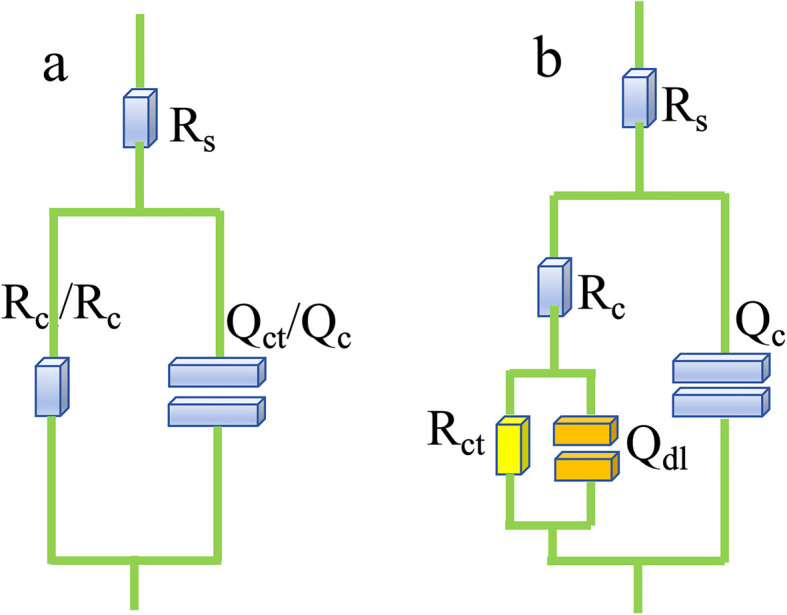
(a) One-time constant R(RQ) and (b) two-time constant R(Q(R(RQ))) models for different samples.

Generally speaking, the capacitive reactance arc of all samples displayed a shrinking trend, which implied that the corrosion resistance was gradually degrading. The time constant in the low frequency and medium frequency regions was attributed to the corrosion response in comparison with the high frequency region, corresponding to the coating response. The largest capacitive semicircle diameter was observed for the Ti_3_C_2_T_*x*_@CoAl-LDH coating, which indicated that the coating had a better corrosion shielding performance. Meanwhile, as shown in Fig. 7, the Nyquist plots for the bare substrate were best fitted using a one-time constant model, R(RQ), corresponding to the charge transfer process at the metal/solution interface. In contrast, the coated samples required a two-time constant model, R(Q(R(RQ))), to accurately represent the dual-layer protective behavior: the first time constant is attributed to the porous outer layer of the hybrid coating, and the second to the charge transfer resistance at the inhibited metal/coating interface.

**Fig. 8:**
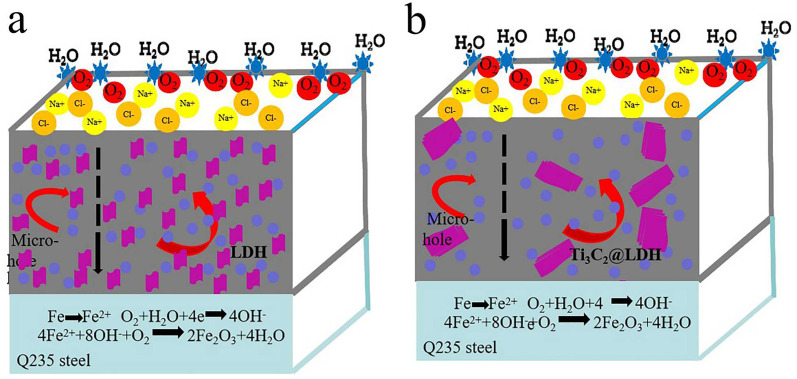
Corrosion protection mechanism of (a) CoAl-LDH and (b) Ti_3_C_2_T_*x*_@CoAl-LDH coatings.

The corrosion protective mechanisms of CoAl-LDH and Ti_3_C_2_T_*x*_@CoAl-LDH coatings are illustrated in Fig. 8^37,38^ The pure CoAl-LDH coating was easily corroded by the corrosive medium due to its low-density structure. The introduction of Ti_3_C_2_T_*x*_ sheets could lengthen the diffusion path of the corrosive media because of their 2D lamellar structure to a certain extent. However, the occurrence of many O groups on the surface of Ti_3_C_2_T_*x*_ makes the absorption of water, O_2_ and other corrosive media easy, which could accelerate the corrosion process.^39^ Interestingly, the two layered materials maintained much stronger interaction with the aggregated structure, which improved the chemical compatibility of Ti_3_C_2_T_*x*_ and CoAl-LDH caused by the synergistic effects between them.^40^ Furthermore, the amino groups intercalated on the Ti_3_C_2_T_*x*_ still maintain a stronger interaction with CoAl-LDH, which improved the chemical compatibility of Ti_3_C_2_T_*x*_ and CoAl-LDH. Thus, good dispersion and compatibility formed an effective barrier network. Due to the dense network, the diffusion path was significantly intricate and the diffusion rate was retarded. Meanwhile, the Ti_3_C_2_T_*x*_@CoAl-LDH exhibited improved hydrophobicity compared with raw CoAl-LDH, which decreased the absorption of water. Consequently, the Ti_3_C_2_T_*x*_@CoAl-LDH composite demonstrates better corrosion resistance performance.^41–43^

The revealed corrosion protective mechanisms of the pure CoAl-LDH and the hybrid Ti_3_C_2_T_*x*_@CoAl-LDH coatings highlight a fundamental shift from a simple physical barrier to a sophisticated, multi-functional defense system. The pure CoAl-LDH coating, while possessing some inherent protective qualities, suffers from intrinsic structural limitations. Its architecture is not entirely dense, featuring micro-pores and interlayer pathways that act as highways for the ingress of corrosive agents such as chloride ions, water molecules, and oxygen. This allows the corrosive electrolyte to readily penetrate and reach the underlying steel substrate, initiating rapid electrochemical reactions like anodic iron dissolution and cathodic oxygen reduction, leading to swift coating failure.^44,45^

The introduction of Ti_3_C_2_T_*x*_ MXene sheets into this system addresses this issue on one front but introduces a challenge on another. Primarily, the two-dimensional, impermeable lamellae of Ti_3_C_2_T_*x*_ act as a superb physical obstacle. When dispersed within the LDH matrix, these nanosheets create a “labyrinth effect,” forcing corrosive media to follow a highly tortuous and elongated diffusion path around them. This significantly delays the time required for the electrolyte to breach the coating and reach the metal interface. However, the inherent surface chemistry of Ti_3_C_2_T_*x*_ presents a paradox. The abundance of hydrophilic functional groups, primarily –O and –OH, on its surface can act as adsorption sites for water and other polar corrosive molecules. This hydrophilicity can potentially create localized reservoirs of electrolyte within the coating, inadvertently accelerating the corrosion process at certain sites and undermining the physical barrier effect.

The true innovation of the Ti_3_C_2_T_*x*_@CoAl-LDH composite lies in the synergistic interactions that overcome this limitation and create a unified, robust network. The two layered materials do not simply coexist; they form a strongly integrated aggregated structure with improved chemical compatibility. This interaction is further enhanced by the presence of amino groups intercalated within the Ti_3_C_2_T_*x*_, which serve as effective coupling agents, forming strong chemical or physical bonds with the layers of CoAl-LDH. This enhanced interfacial bonding is critical. It prevents the delamination of the two phases and ensures a homogeneous dispersion of Ti_3_C_2_T_*x*_ nanosheets throughout the LDH matrix, eliminating weak boundaries and macro-defects.

This good dispersion and compatibility are paramount for the formation of an effective, continuous barrier network. The composite coating evolves from a porous structure into a dense, compact film. Within this dense network, the diffusion path for corrosive species becomes not just longer, but significantly more intricate and constricted, dramatically retarding the diffusion rate and permeability of the coating. Furthermore, this synergistic construction bestows an additional crucial property: enhanced hydrophobicity. The composite surface becomes less amenable to water wetting compared to the raw, hydrophilic CoAl-LDH. This reduced water absorption directly decreases the volume of the electrolyte in contact with the coating, stifling the very medium essential for the corrosion reaction to proceed. Consequently, through the combined action of a tortuous physical barrier, strong interfacial compatibility, and a more hydrophobic surface, the Ti_3_C_2_T_*x*_@CoAl-LDH composite exhibits vastly superior corrosion resistance performance, effectively shielding the steel substrate from the aggressive environment.

## 4. Conclusions

In this work, Ti_3_C_2_T_*x*_@CoAl-LDH nanohybrids were successfully synthesized *via* a rational design strategy. A suite of advanced characterization techniques was systematically employed to unravel the morphologies, structural evolution, and chemical states of the pristine Ti_3_C_2_T_*x*_, CoAl-LDH, and the resulting nanohybrid. The core of this study involved a detailed and rigorous scrutiny of the corrosion resistance properties of the Ti_3_C_2_T_*x*_@CoAl-LDH composite coating. This work successfully underscores the practical application of these novel nanohybrids in advanced anti-corrosion coatings, thereby paving the way for broadening the utilization of Ti_3_C_2_T_*x*_ MXene materials with their unique two-dimensional layered structures beyond energy storage and into the realm of protective surface technologies.

The main results acquired from this comprehensive investigation are succinctly summarized as follows:

(1) The structural and chemical analyses provided unequivocal evidence for the successful formation of the nanohybrid. XRD patterns confirmed the successful preparation of well-crystallized CoAl-LDH. More importantly, the results from FT-IR spectroscopy and SEM-based elemental mapping collectively revealed that Ti_3_C_2_T_*x*_ was effectively modified and integrated with CoAl-LDH. These techniques demonstrated not only the presence of both components but also their intimate interaction, which preserves the essential layered architecture crucial for creating a tortuous barrier pathway.

(2) The electrochemical corrosion evaluation conclusively demonstrated the superior performance of the hybrid coating. In a simulated aggressive environment of NaCl solution, the Ti_3_C_2_T_*x*_@CoAl-LDH coating manifested drastically enhanced corrosion resistance compared to its pure CoAl-LDH counterpart. This is quantitatively highlighted by a remarkably high charge transfer resistance of 1.656 × 10^5^ Ω cm^2^, indicating a formidable barrier against the charge flow necessary for corrosion reactions. Furthermore, the coating delivered outstanding performance with the lowest corrosion current density of 2.833 × 10^−9^ A cm^−2^, which is orders of magnitude lower than that of the pure coating, signifying an exceptionally slow rate of metal dissolution. This synergistic enhancement is attributed to the combined effect of the impermeable Ti_3_C_2_T_*x*_ MXene nanosheets elongating the diffusion path for corrodents and the LDH matrix, forming a dense, compact network that effectively seals defects.

## Conflicts of interest

There are no conflicts to declare.

## Data Availability

All data generated or analysed during this study are included in this published article.

## References

[cit1] Tian Y., Yang Q., Li W., Gong Y., Zhao Q., Li C., Sheng X. (2024). Mater. Adv..

[cit2] Dai W., Yu L. B., Chen Z. Y., Lin C. T., Wang N., Wang H., Ruan K. P., Yu J. H., Yu Z. Z. (2018). ACS Appl. Mater. Interfaces.

[cit3] Li X. L., Shao Y. W., Chen Z. H., Wang Y. Q., Wang J. Y. (2025). Prog. Org. Coat..

[cit4] Islam D. A., Barman K., Jasimuddin Sk., Acharya H. (2019). Nanoscale.

[cit5] Shuai T., Zhan Q., Xu H., Huang C., Zhang Z., Li G. (2023). Chem. Commun..

[cit6] Riazi H., Nemani S. K., Grady M. C., Anasori B., Soroush M. (2021). J. Mater. Chem. A.

[cit7] Kousar K., Walczak M. S., Ljungdahl T., Wetzel A., Oskarsson H., Restuccia P., Ahmad E. A., Harrison N. M., Lindsay R. (2021). Corrosion. Sci..

[cit8] Hang T. T. X., Truc T. A., Olivier M. G., Vandermiers C., Guerit N., Pebere N. (2010). Prog. Org. Coat..

[cit9] Y S., Rao P. (2019). Surf. Interfaces.

[cit10] Kouloumbi N., Tsangaris G. M., Skordos A., Kyvelidis S. (1996). Prog. Org. Coat..

[cit11] Zheng K., Cheng P., Lei L., Wang X., He P., Sun Y., Li J., Jiang Y. (2021). Corros. Sci..

[cit12] Tang Z. (2019). Curr. Opin. Solid State Mater. Sci..

[cit13] Zhang Y., Dou B., Shao Y., Cui X., Wang Y., Meng G., Lin X. (2018). Anti-Corrosion. Methods M..

[cit14] Hu J., Cao S., Xie J., Yin L. (2014). Anti-Corrosion. Methods M..

[cit15] Eynde V., Paradelo R., Monterroso C. (2009). Corrosion. Sci..

[cit16] Attaei M., Calado L. M., Morozov Y., Taryba M. G., Shakoor R. A., Kahraman R., Marques A. C., Montemor M. F. (2020). Prog. Org. Coat..

[cit17] Chong Y., Sun D., Zhang X., Yue C., Yang J. (2019). Chem. Eng. J..

[cit18] Li Z., Yang W., Yu Q., Wu Y., Wang D., Liang J., Zhou F. (2018). Langmuir.

[cit19] Xhanari K., Wang Y., Yang Z., Finsgar M. (2021). Chem. Rec..

[cit20] Zhang Z., Tian N., Huang X., Shang W., Wu L. (2016). RSC Adv..

[cit21] Ramezanzadeh M., Bahlakeh G., Ramezanzadeh B., Sanaei Z. (2019). J. Ind. Eng. Chem..

[cit22] Boudalia M., Guenbour A., Bellaouchou A., Laqhaili A., Mousaddak M., Hakiki A., Hammouti B., Ebenso E. E. (2013). Int. J. Electrochem. Sci..

[cit23] Lin B., Tang J., Wang Y., Wang H., Zuo Y. (2020). Molecules.

[cit24] Dehghani A., Bahlakeh G., Ramezanzadeh B., Mostafatabar A. H., Ramezanzadeh M. (2020). Constr. Build. Mater..

[cit25] Ghaffari S., Aliofkhazraei M., Rouhaghdam A. S. (2019). Prot. Met. Phys. Chem..

[cit26] Zhang W., Li H., Li Y., Ma C., Pan Q., Chen L., Wang L., Wu Y. (2020). J. Mol. Liq..

[cit27] Xu J., Song Y., Zhao Y., Jiang L., Mei Y., Chen P. (2018). Appl. Clay Sci..

[cit28] Olya N., Ghasemi E., Ramezanzadeh B., Mahdavian M. (2020). Surf. Coat. Tech..

[cit29] Shkirskiy V., Keil P., Hintze-Bruening H., Leroux F., Vialat P., Lefevre G., Ogle K., Volovitch P. (2015). ACS Appl. Mater. Inter..

[cit30] Wang T., Cao H., Ma X., Shen X., Min Y., Xu Q. (2024). Corros. Sci..

[cit31] Zhang F., Liu W., Wang S., Liu C., Shi H., Liang L., Pi K. (2021). Composites, Part B.

[cit32] Jee Y. C., Yun J. S., Im S. H., Kim W. S. (2024). Chem. Eng. J..

[cit33] Yan H., Cai M., Li W., Fan X., Zhu M. (2020). J. Mater. Sci. Technol..

[cit34] Benchakar M., Bilyk T., Garnero C., Loupias L., Morais C., Pacaud J., Canaff C., Chartier P., Morisset S., Guignard N., Mauchamp V., celerier S., Habrioux A. (2019). Adv. Mater. Interfaces.

[cit35] Yan L., Zhang B., Wu S., Yu J. (2020). J. Mater. Chem. A.

[cit36] Sun C., Zuo P., Sun W., Xia R., Dong Z., Zhu L., Lv J., Deng G., Tan L., Dai Y. (2020). ACS Appl. Energy Mater..

[cit37] Chavhan J., Rathod R., Umare S., Desai J., Sapate S., Mahajan Y. (2023). Prog. Org. Coat..

[cit38] Lou D., Chen H., Liu J., Wang D., Wang C., Jasthi B. K., Zhu Z., Younes H., Hong H. (2023). ACS Appl. Nano Mater..

[cit39] Asaldoust S., Hosseini M. S., Ramezanzadeh B., Bahlakeh G. (2020). Constr. Build. Mater..

[cit40] Ramezanzadeh M., Ramezanzadeh B., Mahdavian M., Bahlakeh G. (2020). Carbon.

[cit41] Vega J. M., Chimenti S., Lecina E., Grande H., Paulis M., Leiza J. R. (2020). Prog. Org. Coat..

[cit42] Liu X., Wang J., Hu W. (2021). J. Magn. Magn. Mater..

[cit43] Bouali A. C., Andre N. M., Campos M. R., Serdechnova M., Santos J. F., Amancio-Filho S. T., Zheludkevich M. L. (2021). J. Mater. Sci. Technol..

[cit44] Ning Y., Jian D., Liu S., Chen F., Song Y., Li S., Lin B. (2023). Carbon.

[cit45] An D., Wang Z., Qin L., Wu Y., Lu S., Yang H., Ma Z., Mawignon F. J., Liu J., Hao L., Li G. (2023). Prog. Org. Coat..

